# Risk factors for complications in elderly patients aged 85 years and over undergoing endoscopic biliary stone removal

**DOI:** 10.3389/fsurg.2022.989061

**Published:** 2022-10-11

**Authors:** Da-ya Zhang, Ya-qi Zhai, Guan-jun Zhang, Sheng-xin Chen, Lang Wu, De-xin Chen, Ming-yang Li

**Affiliations:** ^1^ Graduate School of PLA General Hospital, Beijing, China; ^2^ Department of Gastroenterology, The First Medical Center, Chinese PLA General Hospital, Beijing, China

**Keywords:** biliary stone, complication, aged, 85 and over, risk factors, cholangiopancreatography, endoscopic retro-grade

## Abstract

**Background and aim:**

The number of elderly patients with biliary stones is increasing. Endoscopic retrograde cholangiography (ERCP) is considered to be an effective treatment for biliary stones. Having a sound knowledge of the risk factors can help reduce the incidence and severity of complications for ERCP. Furthermore, limited research has been published on patients aged over 85 years undergoing endoscopic biliary stone removal. This study aims to determine the risk factors that lead to complications of ERCP in patients over 85 years of age.

**Methods:**

This was a single-center retrospective study. We analyzed 156 patients aged ≥ 85 years with biliary stones who underwent their first ERCP at Chinese PLA General Hospital from February 2002 to March 2021. Logistic regression models were employed to identify the independent risk factors for complications.

**Results:**

A total of 13 patients (8.3%) had complications. Thereinto, pancreatitis, cholangitis, bleeding, and other complications occurred in 4 cases (2.6%), 1 cases (0.6%), 4 cases (2.6%), and 4 cases (2.6%), respectively. There was no perforation or death related to ERCP. Independent risk factors for complications were acute biliary pancreatitis (ABP) (*P* = 0.017) and Charlson Comorbidity Index (CCI) (*P* = 0.019). Significantly, reasons for incomplete stone removal at once were large stone (>10 mm) (*P* < 0.001) and higher acute physiology and chronic health evaluation scoring system (APACHE-II) (*P* = 0.005).

**Conclusions:**

ERCP was recommended with caution in patients ≥ 85 years of age with ABP or higher CCI undergoing endoscopic biliary stone removal. In patients with ABP without cholangitis or biliary obstruction we recommend against urgent (within 48 h) ERCP. Patients with higher CCI who can tolerate ERCP can undergo rapid ERCP biliary stenting or nasobiliary implantation with later treatment of stones, and patients who cannot tolerate ERCP are treated promptly with PTCD and aggressive conservative treatment.

## Introduction

According to a report from the United Nations World Population Prospects, increasing global life expectancy is driving a gradual increase in the proportion of older people (1). The number of elderly patients with biliary stones is also increasing (2). Endoscopic retrograde cholangiopancreatography (ERCP) is an effective treatment for pancreaticobiliary diseases with the aid of x-ray using duodenoscope. Compared with open surgery, ERCP is more acceptable to patients, especially to elderly patients, because of its less invasiveness and faster recovery (3). Although ERCP has many advantages, its postoperative complications should not be ignored, which usually include postoperative pancreatitis, bleeding, perforation, cholangitis, etc., and can lead to death in severe cases (4). ERCP is often thought to be associated with more frequent adverse events (AEs) than other gastrointestinal endoscopic procedures (5). In recent years, ERCP has also been effective and safe in elderly patients ≥65 years of age (6, 7). Complication rates and mortality rates in elderly patients were 6.7%–8.1% and 0%–0.2%, respectively (8–11). However, the complications of ERCP may increase with age, especially in the elderly (12). Having a sound knowledge of the risk factors for ERCP can help reduce the incidence and severity of complications. There are few reports about therapeutic ERCP in elderly patients, and the age stratification is mostly set at 60, 70, or 80 years of age. Furthermore, limited research has been published on patients aged over 85 years undergoing endoscopic biliary stone removal. This study aims to determine the risk factors that lead to complications of ERCP in patients over 85 years of age. In addition, we will explore reasons for incomplete stone removal at one time.

## Materials and methods

### Study design

Data from patients who underwent their first ERCP treatment at Chinese PLA General Hospital from February 2002 to March 2021 were retrospectively analyzed. 156 patients ≥85 years of age or older with biliary stones, including common bile duct stones, Mirizzi syndrome, and intrahepatic bile duct stones, were included for analysis. All patients provided informed consent before ERCP and the protocol was approved by the ethics committees at our hospital (Approval No. S2021-140-01). The study was conducted in accordance with the guidelines of the Declaration of Helsinki.

### Instruments and equipment

Sphincter Incision, Wire Guides, ERCP Catheters, Extraction Ballon, Extraction Basket, Lithotripsy Basket, Biliary Dilation Ballon (Wilson-Cook Medical Incorporated, USA); Nasal Biliary Drainage, Bile Duct Plastic Stent (Cook Ireland Limited, Ireland).

### Surgical method

All patients undergo preoperative laboratory tests such as routine blood tests, coagulation screening and a full set of biochemical tests, and imaging tests such as abdominal Computer Tomography (CT) or Magnetic resonance cholangiopancreatography (MRCP). Patients are fasted for 8 h. Endoscopic procedures were usually conducted under conscious sedation with intravenous diazepam 5.0–10.0 mg and pethidine 50–100 mg. During the procedure, blood pressure, heart rate, respiration, and oxygen saturation are strictly monitored by a multifunctional monitor. Endoscopic ERCP is usually performed first, followed by endoscopic sphincterotomy (EST) and/or endoscopic papillary balloon dilation (EPBD) and/or lithotripsy. If the stone is not removed after the first attempt, endoscopic nasobiliary drainage (ENBD) will be performed and a second attempt will be performed 5–7 days later. If the stone is too large and lithotripsy fails, endoscopic retrograde biliary drainage (ERBD) will be performed. Postoperatively, routine fasting, anti-infection and trypsin inhibitors will be given and abdominal symptoms, signs, blood pressure, temperature, and so on will be closely monitored.

### Outcome and definitions

The primary outcome of this study is the risk factors for ERCP-related complications. The categories and severity of complications are determined according to published criteria (13–15). Pancreatitis was assessed according to the Cotton criteria, with severity defined as mild (elevation of pancreatic enzymes to more than three times the upper limit of normal and prolonged hospital stay of 1–3 days), moderate (prolonged hospital stay of 4–10 days) or severe (prolonged hospital stay of ≥11 days) (13, 14). Cholangitis was diagnosed by the presence of abdominal pain and fever (temperature > 38°C) without any other foci of infection outside the hepatobiliary system and with elevated serum bile enzyme levels compared with pre-ERCP values (14). The severity of cholangitis was assessed according to the Tokyo Guidelines 2018 (15). Other types of AEs were evaluated according to the endoscopic AEs of ASGE workshop (14). Incomplete stone removal is defined as cases wherein bile duct stones could not be removed at once. Comorbidities are defined as diseases that require ongoing medication during the perioperative period of an ERCP or that require modified therapy or medication management. We assess patients' comorbidities using the Charlson Comorbidity Index (CCI), which is widely used as a predictor of mortality for a variety of diseases (16, 17). The index predicts 10-year mortality for 22 different underlying diseases and medical conditions that include heart disease and malignancy. Each condition is assigned a score of 1, 2, 3 or 6 based on the risk of death, and the sum of these scores is used as the total score to predict mortality. ERCP performed during an emergency room stay or on the day of admission is called emergency procedure (18, 19). The degree of the procedural difficulty was classified into 5 categories according to Cotton PB et al. (20).

## Statistical analysis

We used SPSS 27.0 (Armonk, NY: IBM Corp) to analyse the data. Pearson *χ*^2^ test, Two-sample *t*-tests and Mann-Whitney *U* test were used to compare variables between the groups. Odds ratios [OR] were used with 95% confidence limits. *P*-values < 0.05 were considered statistically significant. Factors with a *P*-value < 0.05 by univariate analysis or potentially relevant risk factors based on OR were included in binomial logistic regression analysis to identify independent risk factors for complications and reasons for incomplete stone removal at once.

## Results

The demographics and procedural details were outlined in Tables 1, 2. A total of 13 (8.3%) patients had complications, of which, 9 (5.8%) were mild, 3 (1.9%) were moderate and 1 (0.6%) was severe. Pancreatitis was present in 4 cases (2.6%); mild in 2 cases (1.3%) and moderate in 2 cases (1.3%). Bleeding occurred in 4 cases (2.6%); mild in 4 cases (2.6%). There were no ERCP-related perforations or deaths. Other complications after ERCP were shown in Table 3. Table 4 showed the results of the univariate analysis of risk factors for complications. Univariate analysis showed ABP and CCI were risk factors for complications. For patients who could not tolerate complete stone removal at once, univariate analysis (Table 5) demonstrated that large stones (>10 mm), APACHE-II, American Society of Anesthesiologists (ASA) ≥ 3, emergency ERCP, and multiple large stones were significant risk factors. Independent risk factors for complications were ABP (*P* = 0.017) and CCI (*P *= 0.019) (Table 6). Multi-level logistic regression indicated that the reasons for incomplete stone removal were large stone (>10 mm) and higher APACHE-II (Table 6).

**Table 1:** Characteristics of patients.

Parameter	All patients (*N* = 156)
Female	71 (45.5)
Age, median (IQR)	87 (86–89)
Age ≥90 years	38 (24.4)
Clinical features	
Painless jaundice	4 (2.6)
Cholangitis	113 (72.4)
Abdominal pain with increased liver enzymes	29 (18.6)
ABP	17 (10.9)
others	10 (6.4)
Anticoagulant drug intake	45 (28.8)
Pre-ERCP serum Alb <35 mg/dl	93 (59.6)
Post-cholecystectomy	30 (19.2)
cirrhosis frequency	2 (1.3)
Dialysis frequency	1 (0.6)
Chronic concomitant disease	81 (51.9)
CCI, median (IQR)	1 (0–2)
CCI ≥3	25 (16.0)
ASA ≥3	81 (51.9)
APACHE-II, median (IQR)	5 (5–7)

Data are presented as number (%); ERCP, Endoscopic retrograde cholangiography; ABP, Acute biliary pancreatitis; CCI, Charlson Comorbidity Index; APACHE-II, acute physiology and chronic health evaluation scoring system; ASA, American Society of Anesthesiologists; Alb, albumin; IQR, interquartile range.

**Table 2:** Procedural details of patients treated by ERCP.

Parameter	All patients (*N* = 156)
Emergency ERCP	18 (11.5)
Periampullary diverticulum	74 (47.4)
Cannulation time >10 min (Difficult cannulation)	21 (13.5)
Stone location	
Common bile duct	153 (98.1)
Mirizzi syndrome	2 (1.3)
Intrahepatic	1 (0.6)
Multiple stones	83 (53.2)
Large stone (>10 mm)	97 (62.2)
Multiple large stones	45 (28.8)
Diameter of common bile duct [mean ± SD, (range)], mm	16.8 ± 4.8 (5.8–32)
Dilated common bile duct (>10 mm)	146 (93.6)
Treatment procedure	
Precut	14 (9.0)
Biliary sphincterotomy	130 (83.3)
EST	142 (91.0)
EPBD	20 (12.8)
EL (EHL, EML)	15 (9.6)
ERBD or ENBD	54 (34.6)
Successful cannulation	156 (100)
Complete stone removal	119 (76.3)
Incomplete stone removal	37 (23.7)
Difficulty grade	
1	23 (14.7)
2	56 (35.9)
3	75 (48.1)
4	2 (1.3)
Difficulty grade ≥3	77 (49.4)
Treatment time in minutes, median (IQR)	23 (15–40)

Data are presented as number (%); Endoscopic retrograde cholangiography, ERCP; endoscopic sphincterotomy, EST; endoscopic nasobiliary drainage, ENBD; endoscopic retrograde biliary drainage, ERBD; Endoscopic papillary balloon dilation, EPBD; endoscopic lithotripsy, EL; electrohydraulic shock-wave lithotripsy, EHL; Endoscopic mechanical lithotripsy, EML; interquartile range, IQR.

**Table 3:** Complications of ERCP.

	No. (%)	Severity			
		Mild	Moderate	Severe	Fatal
Overall	13 (8.3)	9 (5.8)	3 (1.9)	1 (0.6)	0 (0)
Pancreatitis	4 (2.6)	2 (1.3)	2 (1.3)	0 (0)	0 (0)
Bleeding	4 (2.6)	4 (2.6)	0 (0)	0 (0)	0 (0)
Perforation	0 (0)	0 (0)	0 (0)	0 (0)	0 (0)
Cholangitis	1 (0.6)	0 (0)	1 (0.6)	0 (0)	0 (0)
Acute heart failure	1 (0.6)	1 (0.6)	0 (0)	0 (0)	0 (0)
Low blood pressure	1 (0.6)	1 (0.6)	0 (0)	0 (0)	0 (0)
Pneumonia	1 (0.6)	0 (0)	0 (0)	1 (0.6)	0 (0)
Dizziness and drowsiness	1 (0.6)	1 (0.6)	0 (0)	0 (0)	0 (0)

Data are presented as number (%).

**Table 4:** Risk factors for complications in the univariate.

Variables	Patients with AE (*N* = 13)	Patients without AE (*N* = 143)	Univariate *P* value	Adjusted odds ratio (95% CI)
Female	5	66	0.595	0.729 (0.228–2.337)
Clinical features				
Cholangitis	9	104	0.787	0.844 (0.246–2.898)
ABP	4	13	0.025	4.444 (1.201–16.448)
Anticoagulant drug intake	3	42	0.633	0.721 (0.189–2.754)
Pre-ERCP serum Alb < 35 mg/dl	5	88	0.114	0.391 (0.122–1.255)
CCI, median (IQR)	2 (0–3)	1 (0–2)	0.018	1.550 (1.077–2.229)
ASA ≥ 3	9	72	0.201	2.219 (0.653–7.535)
APACHE-II, median (IQR)	5 (5–7)	5 (5–7)	0.324	1.130 (0.887–1.439)
Periampullary diverticulum	7	67	0.630	1.323 (0.424–4.133)
Multiple stones	7	76	0.961	1.029 (0.329–3.212)
Large stone (>10 mm)	6	91	0.221	0.490 (0.156–1.535)
Multiple large stones	4	41	0.873	1.106 (0.322–3.792)
Diameter of common bile duct [Mean ± SD, (range)], mm	15.5 ± 4.0 (9.2–21.4)	16.9 ± 4.9 (5.8–32.0)	0.314	0.937 (0.824–1.064)
Cannulation time > 10 min	3	18	0.298	2.083 (0.523–8.294)
EST	13	129	0.999	NS
Balloon	6	57	0.659	1.293 (0.413–4.046)
Basket	7	101	0.217	0.485 (0.154–1.530)
Difficulty grade ≥ 3	6	71	0.809	0.869 (0.278–2.714)
Treatment time in minutes, median (IQR)	33.8 (14.8–40.0)	20.7 (14.6–37.0)	0.996	1.000 (0.973–1.028)

ABP, acute biliary pancreatitis; CCI, Charlson comorbidity index; APACHE-II, acute physiology and chronic health evaluation scoring system; ASA, American society of anesthesiologists; Alb, albumin; IQR, interquartile range; EST, endoscopic sphincterotomy; NS, no significant.

**Table 5:** The reasons for incomplete stone removal in the univariate.

Variables	complete stone removal (*N* = 119)	Incomplete stone removal (*N* = 37)	Univariate *P* value	Adjusted odds ratio (95% CI)
Age ≥ 90 years	31	7	0.380	1.510 (0.602–3.784)
Pre-ERCP serum Alb < 35 mg/dl	68	25	0.261	0.640 (0.294–1.394)
CCI, median (IQR)	1 (0–2)	0 (0–2.5)	0.558	0.922 (0.702–1.210)
ASA ≥3	55	26	0.012	0.364 (0.165–0.803)
APACHE-II, median (IQR)	5 (5–7)	7 (5–9)	<0.001	0.708 (0.585–0.857)
Emergency ERCP	8	10	0.002	0.195 (0.070–0.540)
Multiple stones	61	22	0.384	0.717 (0.339–1.515)
Large stone (>10 mm)	62	35	<0.001	0.062 (0.014–0.270)
Multiple large stones	28	17	0.010	0.362 (0.167–0.784)

ERCP, endoscopic retrograde cholangiography; CCI, Charlson comorbidity index; APACHE-II, acute physiology and chronic health evaluation scoring system; ASA, American society of anesthesiologists; Alb, albumin; IQR, interquartile range.

**Table 6:** Multi-level logistic regression. Analysis of risk factors for overall complication and the reasons for incomplete stone removal.

	*P* value	Adjusted odds ratio (95% CI)
Variables for overall complication		
Female	0.087	0.152 (0.018–1.310)
ABP	0.017	17.284 (1.671–178.760)
CCI, median (IQR)	0.019	1.606 (1.082–2.384)
Variables for reasons for incomplete stone removal		
ASA ≥ 3	0.128	0.359 (0.096–1.344)
APACHE-II, median (IQR)	0.005	1.384 (1.102–1.738)
Emergency ERCP	0.188	2.449 (0.646–9.284)
Large stone (>10 mm)	<0.001	32.708 (5.439–196.699)
Multiple large stones	0.924	1.059 (0.328–3.414)

ERCP, endoscopic retrograde cholangiography; CCI, Charlson comorbidity index; APACHE-II, acute physiology and chronic health evaluation scoring system; ASA, American society of anesthesiologists; Alb, albumin; IQR, interquartile range.

## Discussion

ERCP is an effective diagnostic and therapeutic tool for pancreatobiliary diseases including biliary stone. The rate of complications and mortality in elderly patients are 6.7%–8.1% and 0%–0.2%, respectively (8–11). Our study was in concordance with these figures. Therefore, ERCP was safe and effective in elderly patients ≥ 85 years old undergoing endoscopic biliary stone removal.

In the multivariate analysis, ABP was an independent risk factor for ERCP complications. ABP predisposes patients to fluid loss, metabolic disturbances, hypotension, sepsis and other serious consequences, which often results in an increased risk of post-ERCP complications (21). The concept of early ERCP in ABP originated from observational surgery reports that showed that surgical relief of bile duct obstruction in ABP reduced mortality (22). ASGE recommends ERCP to be performed within 24–48 h of the diagnosis of ABP, but does not specify the timing of ERCP in patients with ABP without concomitant cholangitis (23). However, a meta-analysis showed that early ERCP did not reduce AEs, mortality, and other adverse outcomes in ABP patients without cholangitis compared with conservative treatment (24). Therefore, for patients with ABP without cholangitis or biliary obstruction, we recommend conservative treatment including fluid replacement, pain control, nutritional support and antibiotics.

This study hypothesized that CCI in patients ≥ 85 years old was associated with an increased risk of ERCP-related complications. This was similar to the results of a study that concluded CCI ≥ 2 was independently associated with increased complications or mortality in patients receiving ERCP (25). Since its introduction in 1987, CCI has been widely used as a predictor of mortality from a variety of diseases (16, 26). However, some studies have shown that complications of ERCP are not related to comorbidities in elderly patients (27). We believe there are several reasons for this. First, they use comorbidities that could not quantitatively analyze the severity of comorbidities, rather than CCI. Second, selection bias may have influenced these findings. Patients scheduled to receive ERCP were often excluded for safety reasons due to more comorbidities. The innovative of our study is the use of CCI, currently less used in the prediction of complications of ERCP or other endoscopic procedures. Elderly patients with bile duct stones with a higher CCI have poor general status, and appropriate treatment strategies are important. Patients who cannot tolerate ERCP should be treated promptly with percutaneous biliary drainage (PTCD) and aggressive conservative treatment. PTCD is a standard treatment for biliary drainage (28, 29). We recommended patients who can tolerate ERCP can undergo rapid ERCP biliary stenting or nasobiliary implantation with later treatment of stones. Temporary placement of a biliary stent appears to be an effective treatment for common bile duct stones (30). Current reports show that 2 months of biliary stent placement can partially or completely dissolve large stones without any complications (31). In addition, we believe that the shortening of operation time is the guarantee for the safety of ERCP in elderly patients.

The success rate of common bile duct stone (CBDS) removal can reach 85%–90% through conventional methods such as EPBD, EST and lithotripsy, but there are still 10%–15% of CBDS that are difficult to remove at once, which are called “difficult CBDS” (32). The reasons for difficult CBDS mainly include three situations: firstly, the characteristics of the stone itself, such as stone diameter >15 mm, large number and hard texture; secondly, the presence of anatomical variants, such as duodenal stricture and bile duct stricture; thirdly, the patient can not tolerate endoscopic treatment, such as advanced age with poorer general status (33, 34). Similarly, our study found that large stones and higher APACHE-II were common reasons for ERCP in elderly patients who were unable to remove the bile stones at once. In difficult cases, the ERCP strategy should be decided by a collaborative multidisciplinary team discussion. In the future, more high-quality clinical studies are expected to arrive at refined optimal treatment protocols for different conditions of difficult CBDS.

The limitations of this study are the small sample size and the absence of a control group of patients under 65 years of age in a single-center study, which still needs to be validated by the results of a multicenter study with a large sample. In addition, retrospective studies may have some bias, and prospective studies need to be designed to further validate the results of this study.

In conclusion, ERCP was recommended with caution in patients ≥ 85 years of age with ABP or higher CCI undergoing endoscopic biliary stone removal. In patients with ABP without cholangitis or biliary obstruction we recommend against urgent (within 48 h) ERCP. Patients with higher CCI who can tolerate ERCP can undergo rapid ERCP biliary stenting or nasobiliary implantation with later treatment of stones, and patients who cannot tolerate ERCP are treated promptly with PTCD and aggressive conservative treatment.

## Data Availability

The original contributions presented in the study are included in the article/Supplementary Material, further inquiries can be directed to the corresponding author/s.

## References

[1] United Nations, Department of Economic and Social Affairs, Population Division. World Population Prospects: The 2017 Revision, Key Findings and Advance Tables (2017).

[2] van Erpecum KJ. Gallstone disease. Complication of bile-duct stones: acute cholangitis and pancreatitis. Best Pract Res Clin Gastroenterol. (2006) 20:1139–52. 10.1016/j.bpg.2006.03.012 17127193

[3] Siegel JH Kasmin FE. Biliary tract diseases in the elderly: management and outcomes. Gut. (1997) 41(4):433–5. 10.1136/gut.41.4.433 9391238 PMC1891511

[4] ERCP Group, Chinese Society of Digestive Endoscopology; Biliopancreatic Group, Chinese Association of Gastroenterologist and Hepatologist; National Clinical Research Center for Digestive Diseases. Chinese Guidelines for ERCP (2018). Zhonghua Nei Ke Za Zhi. (2018) 57(11):772–801 (in Chinese). 10.3760/cma.j.issn.0578-1426.2018.11.002 30392234

[5] Talukdar R. Complications of ERCP. Best Pract Res Clin Gastroenterol. (2016) 30(5):793–805. 10.1016/j.bpg.2016.10.007 27931637

[6] Ergin E Oruç N Ersöz G Tekeşin O Özütemiz Ö. Prognosis and risk factors of ERCP pancreatitis in elderly. Sci Rep. (2021) 11(1):15930. 10.1038/s41598-021-95484-8 34354184 PMC8342449

[7] Sugimoto S Hattori A Maegawa Y Nakamura H Okuda N Takeuchi T Long-term outcomes of therapeutic endoscopic retrograde cholangiopancreatography for choledocholithiasis in patients ≥90 years old: a multicenter retrospective study. Intern Med. (2021) 60(13):1989–97. 10.2169/internalmedicine.6478-20 33551408 PMC8313914

[8] Sugiyama M Atomi Y. Endoscopic sphincterotomy for bile duct stones in patients 90 years of age and older. Gastrointest Endosc. (2000) 52:187–91. 10.1067/mge.2000.107285 10922089

[9] Ito Y Tsujino T Togawa O Yamamoto N Isayama H Nakata R Endoscopic papillary balloon dilation for the management of bile duct stones in patients 85 years of age and older. Gastrointest Endosc. (2008) 68:477–82. 10.1016/j.gie.2007.10.066 18760175

[10] Saito H Koga T Sakaguchi M Kadono Y Kamikawa K Urata A Safety and efficacy of endoscopic removal of common bile duct stones in elderly patients ≥90 years of age. Intern Med. (2019) 58(15):2125–32. 10.2169/internalmedicine.2546-18 30996182 PMC6709330

[11] Lu Y Chen L Jin Z Bie LK Gong B. Is ERCP both effective and safe for common bile duct stones removal in octogenarians? A comparative study. Aging Clin Exp Res. (2016) 28(4):647–52. 10.1007/s40520-015-0453-x 26395369

[12] Day LW Lin L Somsouk M. Adverse events in older patients undergoing ERCP: a systematic review and meta-analysis. Endosc Int Open. (2014) 2(1):E28–36. 10.1055/s-0034-1365281 26134610 PMC4423280

[13] Cotton PB Lehman G Vennes J Geenen JE Russell RC Meyers WC Endoscopic sphincterotomy complications and their management: an attempt at consensus. Gastrointest Endosc. (1991) 37(3):383–93. 10.1016/S0016-5107(91)70740-2 2070995

[14] ASGE Standards of Practice Committee, Chandrasekhara V Khashab MA Muthusamy VR Acosta RD Agrawal D Bruining DH Adverse events associated with ERCP. Gastrointest Endosc. (2017) 85(1):32–47. 10.1016/j.gie.2016.06.051 27546389

[15] Miura F Okamoto K Takada T Strasberg SM Asbun HJ Pitt HA Tokyo Guidelines 2018: initial management of acute biliary infection and flowchart for acute cholangitis. J Hepatobiliary Pancreat Sci. (2018) 25(1):31–40. 10.1002/jhbp.509 28941329

[16] Charlson ME Pompei P Ales KL MacKenzie CR. A new method of classifying prognostic comorbidity in longitudinal studies: development and validation. J Chron Dis. (1987) 40:373–83. 10.1016/0021-9681(87)90171-8 3558716

[17] de Groot V Beckerman H Lankhorst GJ Bouter LM. How to measure comorbidity: a critical review of available methods. J Clin Epidemiol. (2003) 56:221–9. 10.1016/S0895-4356(02)00585-1 12725876

[18] Shah SL Carr-Locke D. ERCP For acute cholangitis: timing is everything. Gastrointest Endosc. (2020) 91(4):761–2. 10.1016/j.gie.2019.12.010 32204811

[19] Iqbal U Khara HS Hu Y Khan MA Ovalle A Siddique O Emergent versus urgent ERCP in acute cholangitis: a systematic review and meta-analysis. Gastrointest Endosc. (2020) 91(4):753–760.e4. 10.1016/j.gie.2019.09.040 31628955

[20] Cotton PB Eisen G Romagnuolo J Vargo J Baron T Tarnasky P Grading the complexity of endoscopic procedures: results of an ASGE working party. Gastrointest Endosc. (2011) 73(5):868–74. 10.1016/j.gie.2010.12.036 21377673

[21] Forsmark CE Vege SS Wilcox CM. Acute pancreatitis. N Engl J Med. (2016) 375(20):1972–81. 10.1056/NEJMra1505202 27959604 PMC13220086

[22] Acosta JM Rossi R Galli OM Pellegrini CA Skinner DB. Early surgery for acute gallstone pancreatitis: evaluation of a systematic approach. Surgery. (1978) 83:367–70. PMID: 635772

[23] ASGE Standards of Practice Committee, Buxbaum JL Abbas Fehmi SM Sultan S Fishman DS Qumseya BJ Cortessis VK ASGE Guideline on the role of endoscopy in the evaluation and management of choledocholithiasis. Gastrointest Endosc. (2019) 89(6):1075–1105.e15. 10.1016/j.gie.2018.10.001 30979521 PMC8594622

[24] Shrestha DB Budhathoki P Sedhai YR Adhikari A Poudel A Aryal BB Urgent endoscopic retrograde cholangiopancreatography (ERCP) vs. conventional approach in acute biliary pancreatitis without cholangitis: an updated systematic review and meta-analysis. Cureus. (2022) 14(1):e21342. 10.7759/cureus.21342 35198265 PMC8852244

[25] Tabak F Wang HS Li QP Ge XX Wang F Ji GZ Endoscopic retrograde cholangiopancreatography in elderly patients: difficult cannulation and adverse events. World J Clin Cases. (2020) 8(14):2988–99. 10.12998/wjcc.v8.i14.2988 32775380 PMC7385608

[26] Mnatzaganian G Ryan P Norman PE Hiller JE. Accuracy of hospital morbidity data and the performance of comorbidity scores as predictors of mortality. J Clin Epidemiol. (2012) 65:107–15. 10.1016/j.jclinepi.2011.03.014 21803545

[27] Han SJ Lee TH Kang BI Choi HJ Lee YN Cha SW Efficacy and safety of therapeutic endoscopic retrograde cholangiopancreatography in the elderly over 80 years. Dig Dis Sci. (2016) 61:2094–101. 10.1007/s10620-016-4064-y 26873537

[28] Verma N Hema HK Gupta P Kang M Kalra N Samanta J Role of percutaneous transhepatic biliary drainage as an adjunct to endoscopic retrograde cholangiopancreatography. J Clin Exp Hepatol. (2022) 12(2):287–92. 10.1016/j.jceh.2021.09.002 35535076 PMC9077227

[29] Hayat U Bakker C Dirweesh A Khan MY Adler DG Okut H EUS-guided versus percutaneous transhepatic cholangiography biliary drainage for obstructed distal malignant biliary strictures in patients who have failed endoscopic retrograde cholangiopancreatography: a systematic review and meta-analysis. Endosc Ultrasound. (2022) 11(1):4–16. 10.4103/EUS-D-21-00009 35083977 PMC8887045

[30] Bergman J Rauws EAJ Tijssen JGP Tytgat GN Huibregtse K. Biliary endoprostheses in elderly patients with endoscopically irretrievable common bile-duct stones—report on 117 patients. Gastrointest Endosc. (1995) 42:195–201. 10.1016/S0016-5107(95)70091-9 7498682

[31] Horiuchi A Nakayama Y Kajiyama M Kato N Kamijima T Graham DY Biliary stenting in the management of large or multiple common bile duct stones. Gastrointest Endosc. (2010) 71(7):1200–3. 10.1016/j.gie.2009.12.055 20400079

[32] Ryozawa S Itoi T Katanuma A Okabe Y Kato H Horaguchi J Japan Gastroenterological endoscopy society guidelines for endoscopic sphincterotomy. Dig Endosc. (2018) 30(2):149–73. 10.1111/den.13001 29247546

[33] Manes G Paspatis G Aabakken L Anderloni A Arvanitakis M Ah-Soune P Endoscopic management of common bile duct stones: european society of gastrointestinal endoscopy (ESGE) guideline. Endoscopy. (2019) 51(5):472–91. 10.1055/a-0862-0346 30943551

[34] Aburajab M Dua K. Endoscopic management of difficult bile duct stones. Curr Gastroenterol Rep. (2018) 20(2):8. 10.1007/s11894-018-0613-1 29572696

